# Faculty commitment, effectiveness of job responsibilities and the moderating role of institutional support: A survey data set

**DOI:** 10.1016/j.dib.2018.05.138

**Published:** 2018-05-31

**Authors:** H.O. Falola, O.A. Oludayo, D.M. Akinnusi, A.O. Osibanjo, O.P. Salau

**Affiliations:** Department of Business Management, Covenant University, Ota, Nigeria

## Abstract

The main objective of this paper is to present the data set which depicts faculty commitment and effectiveness of job responsibilities in a changing world and the moderating role of the university׳s support system. The population of the study consisted all the 1912 Faculty members of six selected private universities in Southwest, Nigeria [5]. The sample size determination formula by [5] was adopted, resulting in the selection of 400 respondents to whom a structured questionnaire was administered accordingly. Only 343 copies of the questionnaire were valid and used for this data set. Structural equation modeling, which combines factor analysis and multiple regression, was used to present the structural relationship between dependent and independent variables. When the data is analysed, it will help to determine the degree of relationship and the strength of significance between the observed variables and the latent constructs.

**Specification Table**

**Table:** 

**Subject area**	Management and Higher Education
**More Specific Subject Area**	Human Resource Management and Industrial Relations
**Type of Data**	Table
**How Data was Acquired**	Through the administration of a structured questionnaire to Faculty members
**Data format**	Raw, analyzed and statistical data
**Experimental Factors**	Stratified and simple random sampling of Faculty members of some selected private universities in Southwest Nigeria.
**Experimental features**	The perception of faculty members on the influence of job commitment on the effectiveness of job responsibilities.
**Data source location**	South west Nigeria
**Data Accessibility**	All the data are included in this article

**Value of the data**

• The data covers a representative sample of private universities in Nigeria, thus enhancing external validity of the findings.
• University management can leverage on the data, if analysed, for the purpose of decision making regarding employee commitment and job responsibilities
• The analysis of this data can give valuable insights into the roles which university support plays in enhancing job commitment and responsibilities. See [1], [3], [7] for similar data.
• The data can be used as a platform for further investigation by other researchers.
• The data provided here can be used for educational and change management purposes.
• The research instrument can be adopted or adapted for similar studies
• This data can be used to determine the unique dimensions of relationships and significant effects of faculty commitment, university support and effectiveness of job responsibilities of faculty members.

## 1 Data

**Fig. 1:**
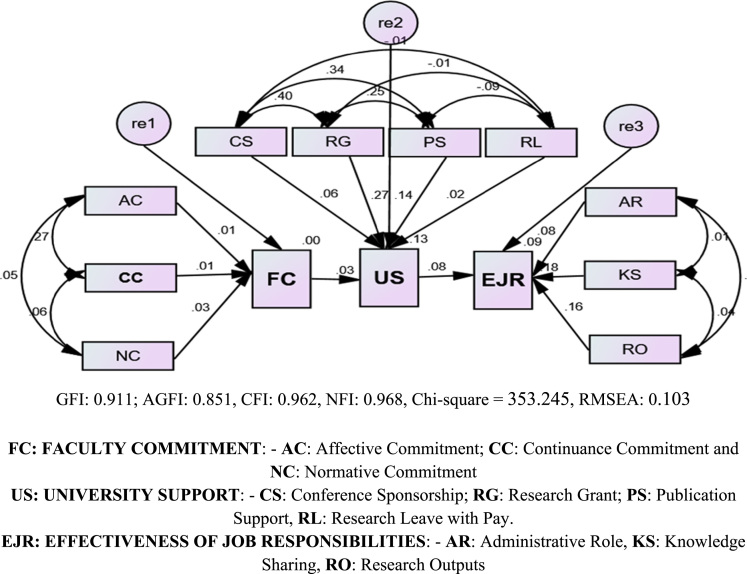
Faculty Commitment, University Support and Effectiveness Job Responsibilities Model.

The data comprised raw statistical data on the influence of faculty commitment on the effectiveness of job responsibilities, with the role of the university system as a moderating factor. Descriptive design was adopted for this data set. Statistical Package for Social Sciences (SPSS) and AMOS 22. Structural Equation Modelling (SEM) was used to determine the strength of relationship and resultant effects of the observed variables and the latent constructs. Fig. 1 and Table 1 shows the output of structural equation modeling and the regression weight of the data processed. The processed data for the study were gotten through the use of the 5-point Likert scale [2], [6]. The analysis of this data can provide a deep insight into what the university management should do to earn the commitment of faculty members and enhance their job responsibilities particularly in this contemporary and highly competitive academic environment.

**Table 1:** Regression weights

			Estimate	S.E.	C.R.	P
FC	<---	AC	.008	.102	.177	.859
FC	<---	CC	.014	.103	.318	.751
FC	<---	NC	.029	.092	.658	.510
US	<---	CS	.060	.040	1.289	.197
US	<---	RL	.024	.041	.586	.558
US	<---	RG	.265	.045	5.886	***
US	<---	PS	.136	.019	2.977	.003
US	<---	FC	.031	.019	.736	.462
EJR	<---	US	.082	.042	1.918	.055
EJR	<---	AR	.085	.043	1.694	.090
EJR	<---	RO	.158	.047	3.164	.002
EJR	<---	KS	.181	.038	4.255	***

## 2 Experimental design, materials and methods

In order to determine the sample size, [1], [5] formula was used

$$n=\frac{N}{\Sigma \left[1+N{\left(e\right)}^{2}\right]}$$

Where;

**n** = the desired sample size to be determined
**N** = total population
**e** = accepted error limit (0.05) on the basis of 95% confidence level.

N=1912e=0.05n=samplesize

$\begin{array}{c} n=\frac{1912}{1+1912\left(0.05\right)2}\\ n=\frac{1912}{1913\left(0.0025\right)}\\ n=\frac{1912}{4.7825}\\ n=400 \end{array}$

The data presented above was based on the quantitative study. To investigate the effect of faculty commitment on the effectiveness of job responsibilities, survey research design was adopted. The best six private universities in Southwest Nigeria were sampled (2,7). The study population consisted all the ranks of the 1912 faculty members of the selected universities. Four hundred (400) of the faculty members were selected across all colleges to participate in this study with the use of a structured questionnaire. However, information on the details of the study population can equally be accessed in [3]. Data was analyzed using structural equation modeling (AMOS 22) and the outputs provide in-depth insights into what the management of universities should be doing to enhance faculty commitment to their job responsibilities. More information on the use of structural equation model and AMOS can be accessed in [3], [4], [7]. The researchers ensured that management of the selected universities were adequately informed about the objective of the study and the permission was sought and granted for the administration of questionnaire to the Faculty members. In addition, Faculty members were equally informed about the objective of the study and as well assured that their responses will be treated with topmost confidentiality.

The data provides insight into the role institutional support plays in enhancing Faculty commitment and effectiveness of job responsibilities. The data presented in this article may assist the management of the institutions of higher learning to have deep insight and understanding into the significant role of institutional support in enhancing faculty commitment and effectiveness of job responsibilities. This suggests that management of the selected universities may leverage on the data for the purpose decision making, educational and change management purposes. The data presented could also be used for further investigation.
