# Supplementary material for: Faculty commitment, effectiveness of job responsibilities and the moderating role of institutional support: A survey data set
Source: Data Brief. 2018 May 31;19:1120–3. doi: 10.1016/j.dib.2018.05.138 (PMC6141761; doi:10.1016/j.dib.2018.05.138)
Supplement: Supplementary file 1 — Supplementary material [file mmc1.doc]

**Falola Hezekiah Olubusayo (Ph.D)**

Department of Business Management

College of Business and Social Sciences,

Covenant University, Ota, Ogun State, Nigeria

[hezekiah.falola@covenantuniversity.edu.ng](mailto:hezekiah.falola@covenantuniversity.edu.ng)

+234 703 5518 559

**May 3, 2017**

The Editor,

Data in Brief

Dear Sir,

**DECLARATION OF CONFLICT OF INTEREST**

I, Dr. Falola H.O and my colleagues write to declare that there is no conflict of interest traceable to our data paper “Faculty commitment, effectiveness of job responsibilities and the moderating role of institutional support: A survey data set”

Thank you.

Yours faithfully,


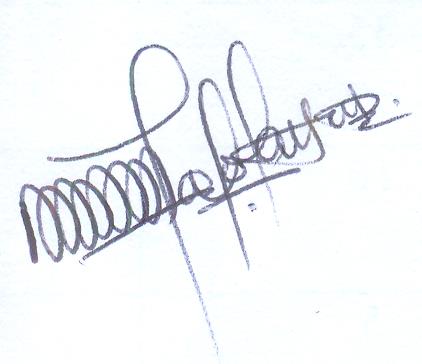


FALOLA H.O (PhD)

**Corresponding Author**
